# Start a Neonatal Extracorporeal Membrane Oxygenation Program: A Multistep Team Training

**DOI:** 10.3389/fped.2018.00151

**Published:** 2018-05-29

**Authors:** Genny Raffaeli, Stefano Ghirardello, Mara Vanzati, Chiara Baracetti, Francesco Canesi, Federica Conigliaro, Valerio Gentilino, Francesco Macchini, Monica Fumagalli, Fabrizio Ciralli, Nicola Pesenti, Sofia Passera, Simona Neri, Stefania Franzini, Ernesto Leva, Laura Plevani, Fabio Mosca, Giacomo Cavallaro

**Affiliations:** ^1^Neonatal Intensive Care Unit, Department of Clinical Sciences and Community Health, Fondazione IRCCS Ca' Granda Ospedale Maggiore Policlinico, Università degli Studi di Milano, Milan, Italy; ^2^Betamed Perfusion Service, Rome, Italy; ^3^Department of Pediatric Surgery, Ospedale Filippo Del Ponte Varese, Varese, Italy; ^4^Department of Pediatric Surgery, Fondazione IRCCS Ca' Granda Ospedale Maggiore Policlinico, Milan, Italy; ^5^Pediatric Anesthesiology and Intensive Care Unit, Department of Anesthesia and Critical Care, Fondazione IRCCS Ca' Granda Ospedale Maggiore Policlinico, Milan, Italy

**Keywords:** neonatal ECMO, extracorporeal life support ECLS, ECLS program development, high-fidelity simulation, teamwork, skill learning, ECMO Team

## Abstract

**Background:** Extracorporeal membrane oxygenation (ECMO) is a complex life-saving support for acute cardio-respiratory failure, unresponsive to medical treatment. Emergency events on ECMO are rare but require immediate and proficient management. Multidisciplinary ECMO team members need to acquire and maintain over time cognitive, technical and behavioral skills, to safely face life-threatening clinical scenarios.

**Methods:** A multistep educational program was delivered in a 4-year period to 32 ECMO team members, based on guidelines from the Extracorporeal Life Support Organization. A first traditional module was provided through didactic lectures, hands-on water drills, and laboratory animal training. The second phase consisted of a multi-edition high-fidelity simulation-based training on a modified neonatal mannequin (SimNewB®). In each session, participants were called to face, in small groups, ten critical scenarios, followed by debriefing time. Trainees underwent a pre-test for baseline competency assessment. Once completed the full training program, a post-test was administered. Pre- and post-test scores were compared. Trainees rated the educational program through survey questionnaires.

**Results:** 28 trainees (87.5%) completed the full educational program. ECMO staff skills improved from a median pre-test score of 7.5/18 (IQR = 6–11) to 14/18 (IQR = 14–16) at post-test (*P* < 0.001, Wilcoxon rank test). All trainees highly rated the educational program and its impact on their practice. They reported high-fidelity simulations to be beneficial to novice learners as it increased self-confidence in ECMO-emergencies (according to 100% of surveyed), theoretical knowledge (61.5%) and team-work/communicative skills (58%).

**Conclusions:** The multistep ECMO team training increased staff' knowledge, technical skills, teamwork, and self-confidence, allowing the successful development of a neonatal respiratory ECMO program. Conventional training was perceived as relevant in the early phase of the program development, while the active learning emerged to be more beneficial to master ECMO knowledge, specific skills, and team performance.

## Introduction

Extracorporeal membrane oxygenation (ECMO) is a life-saving, high-risk support for acute cardio-respiratory failure, unresponsive to conventional treatment (1, 2). About 600 neonates require respiratory ECMO worldwide each year (3, 4). Despite considerable advances in equipment and technology, the management of a neonatal respiratory ECMO procedure remains challenging, in spite of the adoption of standardized protocols and preventive strategies. Patients' outcome is still jeopardized by both clinical and technical life-threatening complications, and the overall survival rate is 83% until decannulation, and 72% until discharge, without significant differences over time (4).

Proficiency in a high-tech procedure requires extensive training, which includes comprehensive knowledge of ECMO principles and the technical ability to perform a time-critical and well-coordinated emergency troubleshooting (5). As neonatal respiratory ECMO is a low-frequency procedure, the ECMO team needs to develop cognitive, technical, and behavioral skills in a sustainable way (2). Clinical simulations provide the opportunity to reproduce scenarios “close to real-life” settings (6).

Effective training becomes even more crucial during the developmental process of a neonatal ECMO center (7, 8). As the quality of care is dependent on the ECMO specialists' expertise, their training is fundamental for the implementation and sustainability of a novel ECMO program (8). Traditional ECMO training programs consist of didactic lectures, “water drills” (ECMO circuits training) and animal laboratories. They are mainly based on passive learning (5) whose effectiveness is questioned by some authors (9, 10). In fact, trainees benefit more from active learning, by applying their knowledge, experience, behavioral and technical skills to solve issues (11). Simulation-based training allows learners to focus not only on cognitive aspects but also on critical thinking, behavioral response to emergency situations, teamwork, and effective communication in real-life scenarios (12). The relevance of human factors training has been highlighted by the *crew resource management* (CRM), developed by NASA and aviation industry (13). Mannequin-based neonatal ECMO training was first introduced in 2006 (14), though the relevance of mannequin-based training during the initiation and maintenance of an ECMO program (8, 15, 16) has not been included in the Extracorporeal Life Support Organization training guidelines. Single institutions self-tailor their educational curriculum, resulting in a wide heterogeneity of training practices and a suboptimal implementation of mannequin-based education among ECMO centers (17). Previous experience of comprehensive training implementation has been reported in adults and pediatric ECMO (18), while data on *Neonatal Intensive Care Unit* settings are still limited.

We have developed an in-house neonatal respiratory ECMO program, with no prior *Extracorporeal life support* experience or cardiac surgery back-up. To introduce this sophisticated technology, we have developed an educational curriculum to train neonatologists, pediatric surgeons, nurses, and perfusionists involved in the program.

Here we report our experience to deliver a multistep ECMO training program, and the effectiveness of this educational strategy, as perceived by the trainees.

## Materials and methods

The study was approved by the local Ethics Committee (Milano Area 2, Italy). All subjects gave written informed consent in accordance with the Declaration of Helsinki.

A multistep educational ECMO curriculum was delivered during a 4-year period for 32 ECMO team members, based on guidelines from the ELSO (16). A first module was provided through didactic lectures, hands-on water drills, and laboratory-based animals training. The second phase consisted of a multi-edition, high-fidelity simulation-based training. Besides the in-house training program, both bedside training and attendance at educational “hands-on” courses hosted by established ECMO centers has been guaranteed. The successful completion of the training program was required before the trainees could perform on real patients.

### ECMO program

**Figure 1:**
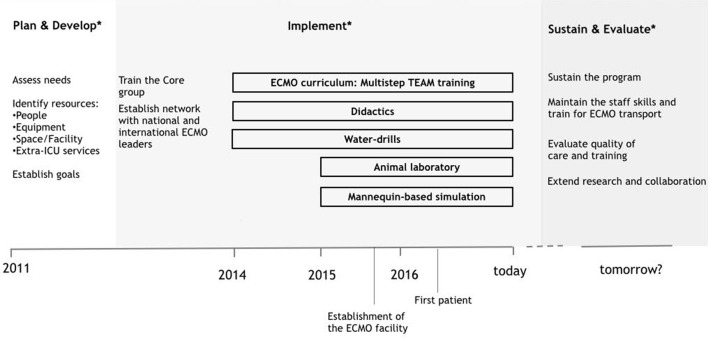
Development of a neonatal respiratory ECMO program: timeline of key steps. *The developmental process is based on ELSO guidelines (8, 16).

The neonatal respiratory ECMO facility has been established at our institution since September 2015. Our *Neonatal Intensive Care Unit* admits about 450 neonates per year. It is a national referral center for fetal and neonatal surgery with an average of 15 congenital diaphragmatic hernias treated each year. The ECMO program follows the “dual provider” model, the critical care nurse takes care of the patient, and the cardiovascular perfusionist is responsible for the ECMO circuit management (8). According to this model, the ECMO physician oversees the entire coordination of care. The training process for the ECMO core group started in 2012 with the delivery of educational material and the attendance to national and international courses and observership programs at ECMO referral centers. Since 2014 a formal curriculum has been extended to all the members of the team. Figure 1 shows the key steps in the developmental process.

### Traditional education

The *didactic course* included 12 lessons (48 h), covering the main clinical aspects of respiratory ECMO: physiology, clinical indications, support modalities (both veno-venous and veno-arterial), equipment, cannulation strategies, coagulation management during the entire ECMO procedure, pharmacotherapy, mechanical and clinical complications, weaning, follow-up, ethics, and ECMO withdrawal. In-house and national trainers were invited for lectures on specific topics. Teaching resources included the ELSO guidelines, the “ELSO Redbook” and the “ELSO ECMO Specialist Training Manual.”

The *Wet labs or water drills* included hands-on training on closed-loop ECMO circuit filled with water, used to address technical issues and to familiarize the practitioners with equipment and procedures (circuit assembly, change, and repair).

**Figure 2:**
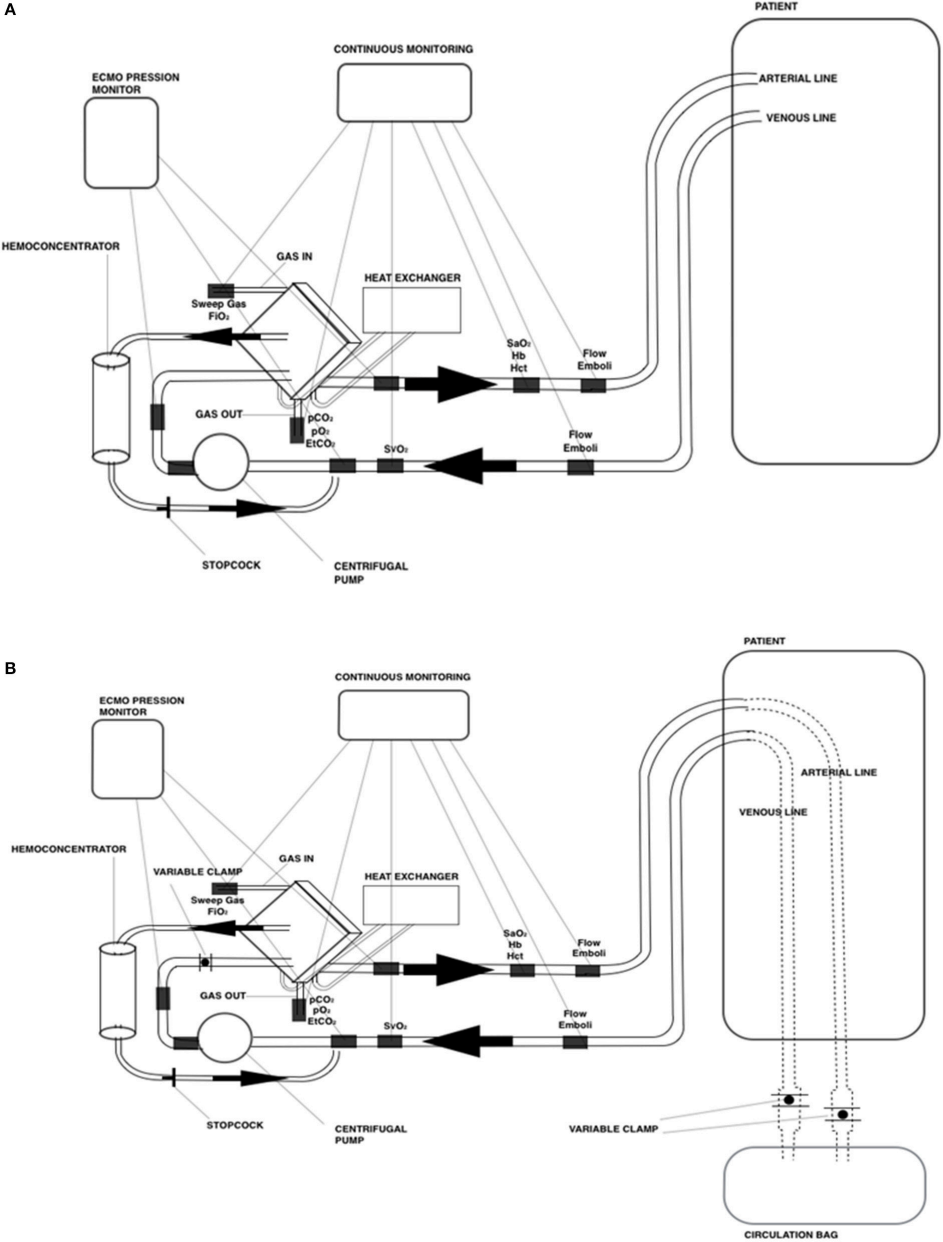
Schematic drawing of the ECMO circuit used for **(A)** animal laboratory and **(B)** mannequin-based simulations.

The *animal laboratory* consisted of establishing a veno-arterial ECMO on anesthetized cannulated piglets and reproducing a daily ECMO management (Figure 2A). During the 8-h ECMO run, 5–8 members of the team, including pediatric surgeons, were directly involved in routine practices (cannulation, blood gas sampling, analysis and interpretation, coagulation management, and weaning).

### High fidelity simulation training

**Figure 3:**
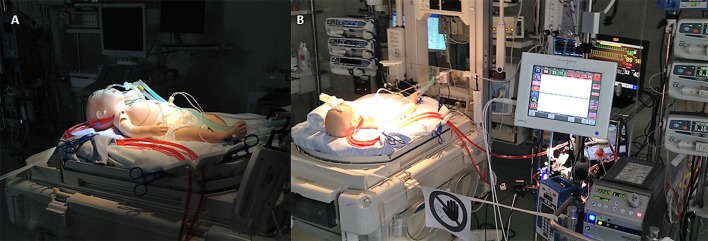
Modified mannequin for ECMO simulation. **(A)** Particular of the cannulas. **(B)** HiFi simulation setting.

We conducted high-fidelity simulation sessions in a dedicated training area at our Institution. Our mobile simulation set-up included a modified high fidelity neonatal mannequin (SimNewB® Laerdal) with an *Extracorporeal life support* bypass (Figures 2B, 3). Tubes were inserted in the right side of the neck of the mannequin and distally connected to a reservoir bag, filled with fake blood. This system allowed the trainers to change hemodynamic parameters either by changing volemia (two ports were placed near the reservoir for fluid administration or withdrawal) or by reducing the tube diameters with clamps. Air entry emergency was simulated by injecting air bubbles through distally placed ports. A white shield allowed instructors to hide their actions to trainees. The ECMO equipment consisted of a centrifugal blood pump, a polymethylpentene membrane oxygenator, flowmeters, ¼ circuit tubing, oxygen flow regulator, heater unit and circuitry non-invasive monitoring (*in vitro* pressures, saturation, and air bubbles). The software for SimNewB® Laerdal was used to remotely plan clinically relevant scenarios and simulate changes of vital signs. The features of simulations adhere to the principles of best educational practice (19). The training room reproduced the real-life *Neonatal Intensive Care Unit* setting with the same equipment (ventilator, supplies, emergency cart, and drugs).

During each session, 3 participants at a time were faced with 10 critical scenarios. Attention was paid to both technical and behavioral factors that would impact patient's outcome. Our team configured the mannequin, the simulation setting, and the scenarios based on veno-venous or veno-arterial modality. The plots were derived from typical ECMO emergency cases and aimed at reaching specific learning outcomes (Table 1). Trainers led the progression of the simulation in real time, according to trainees' reactions. When specific actions (chest drain placement, extracorporeal support changes, etc.) were needed, they were asked to notify their intentions loudly. Critical activities were identified and deemed necessary for the scenario resolution (Table 1). Each scenario ended with extensive debriefing and immediate feedback.

**Table 1:** Simulation-based scenarios.

**Scenario**	**Learning outcome**	**Action**	**Critical action**
Accidental decannulation	ECMO Emergency management Technical skills Teamwork	Evaluate circuit and patient parameters	Provide prompt Backup ventilatory support Patient stabilization
Air in the circuit	ECMO Emergency management Circuit check Technical skills	Recognize presence of air (micro or macro bubbles, massive air) Identify leak source Chest X-ray to confirm cannula position	Cross-clamp (if air is post-oxy or if massive air) Prompt Backup ventilatory support (until complete air removal) Remove air (in all cases)
Clots in the membrane	Routine ECMO management Hemostasis evaluation Teamwork	Try to identify the clot Evaluate trans-membrane delta pressure Order coagulation tests Discuss with hematologist	Check anticoagulation strategy Circuit replacement
Oxygenator failure	Routine ECMO management Technical skills	Gas exchange evaluation (pre- post- membrane)	Discuss need for circuit replacement, based on clinical data
Blood recirculation	Routine ECMO management	Recognize saturation decrease with increasing blood flow Order Echocardiogram Chest X-Ray	Reduce flow Fix the cannula position
Circuit line kinking	Routine ECMO management Circuit check, pressure, and flow evaluation	Recognize kinking Identify possible cause of kinking and establish preventive measures	Provide line patency
Cannula kinking	Routine ECMO management Circuit pressure evaluation Teamwork	Order Echocardiogram Chest X-Ray Call surgeon	Increase ventilator's setting Cannula replacement
Hypovolemia	Routine ECMO patient management Circuit pressure evaluation	Order laboratory tests, echocardiogram	Fluid administration
Bleeding	Surgical emergency Hemostasis evaluation Circuit pressure evaluation Teamwork	Recognize presence and cause of bleeding Coagulation test	Stop the bleeding Blood administration
Pneumothorax	Patient emergency management Circuit pressure evaluation Teamwork	Order Echocardiogram Chest X-Ray Gas exchange	Pneumothorax decompression Patient stabilization

*Outline of related learning outcomes, actions, and critical actions*.

### Measurements and knowledge assessment

We collected demographic data of participants (age, gender, years of experience as a neonatal care provider, previous professional experience in ECMO) and subjected them to a written pre-curriculum test for baseline competency assessment. Once completed the training program, a written post-test with an 18-items checklist was administered (Supplementary Material). All pre- and post-tests were scored by a single instructor, following a dichotomous scale for each question (right or wrong). The final score (X/18) was made by the number of correct answers (X), in relation to the totality (18).

All participants were asked to provide their perception of the educational programme. Trainees rated the educational program using a survey, based on a 5-point Likert scale, from one (strongly disagree) through five (strongly agree). We evaluated each step of the educational program: didactic sessions, wet labs, animal labs, simulation and debriefing sessions; inquiries were focused on the teaching methodology, i.e., knowledge, procedural confidence, teamwork, and real-life fidelity. Participants were given the opportunity to provide open-ended feedback to improve the educational curriculum. The research team designed the survey for the present study.

### Data analysis

We provided a descriptive analysis of the demographic aspects of the participants. Continuous variables that follow an approximately normal distribution were reported as mean (and standard deviation), skewed continuous variables will be summarized using medians and inter-quartile ranges and categorical variables as overall counts and percentage. We compared the average number of correct answers between pre- and post- “curriculum test” with the Wilcoxon rank test; a *p* < 0.05 was considered significant. Statistical analyses were performed using R version 3.4.3 (R Foundation for Statistical Computing, Vienna, Austria).

## Results

During the study period, we provided a total of 12 didactic lectures, 24 “wet labs,” 12 animal laboratories and six high fidelity simulations. 28 trainees (87.5%) completed the full educational program, by attending at least 60% of didactic lectures and one of each practice session. Four trainees (12.5%) did not complete the training for non-compliance with the full schedule. Table 2 showed the demographic data of the participants. ECMO staff skills improved from a median pre-test score of 7.5/18 (IQR = 6–11) to a median post-test score of 14/18 (IQR = 14–16) (*P* < 0.001, Wilcoxon rank test). A focused analysis of the individual questions shows the performance of the ECMO team in relation to every single item (Table 3). In 3 questions (n°8-12-16) the rate of correct answers in the pre-test is below 20%, with an improvement in the post-test, except for one (n°16). In 3 questions (n°3-15-18) the rate of correct answers in the pre-test is over 70%. In 4 questions (n°6-7-9-16) the rate of correct answers remains below 65% at the post-test.

**Table 2:** Demographic data of trainees undergoing ECMO educational program.

**Characteristic**	**Mean (±*SD*) or No. (%)**
Age (years)	38.29 (±7.59)
Male	9 (32.1%)
Role	
Physicians	10 (35.7%)
Nurses	18 (64.3%)
Years of NICU clinical experience	3 (10.7%)
< 5	10 (35.7%)
5–10	8 (28.5%)
11–15	7 (25%)
>15	
Prior ECMO experience or training	2 (7.1%)

*ECMO, extracorporeal membrane oxygenation; NICU, neonatal intensive care unit*.

**Table 3:** Focused analysis of the performance of the ECMO team on individual questions.

**Question (n°)**	**Pre (right answers, %)**	**Post (right answers, %)**	**Improvement (delta, %)**
1	44	94	50
2	61	83	22
3	77	88	11
4	66	72	6
5	55	77	22
6	27	55	28
7	33	61	28
8	11	77	66
9	44	61	17
10	39	67	28
11	39	83	44
12	17	100	83
13	33	89	56
14	44	78	34
15	72	94	22
16	16	50	34
17	67	100	33
18	83	100	17

All trainees have highly rated the educational program and its impact on their practice. They reported high fidelity simulations to be beneficial, as it increased self-confidence in ECMO-emergencies (according to 100% of surveyed), theoretical knowledge (61.5%) and team-work/communicative skills (58%). Results are presented in Table 4.

**Table 4:** ECMO “curriculum” evaluation.

**Survey item**	**Median^(a)^**	**IQ 25°^(a)^**	**IQ 75°^(a)^**
Didactic sessions are beneficial to ECMO learners, to acquire and refresh the theoretical basis.	4	4	5
“Water drills” lab is useful to enhance technical skills and to familiarize with equipment	4	3	5
Animal model adds “real life” aspects, increasing teamwork and communicative skills.	4	3	5
High-fidelity simulation improves my performance skills and my self-confidence. Environment is realistic.	4	3	5
I receive useful educational feedback from the debriefing sessions.	4	4	4
I prefer high-fidelity mannequin to animal-based training.	4.5	4	5
I would recommend the multistep training to be delivered in the same way: theory (didactic lectures) followed by practice on circuits (water drills), on animals (lab) and on neonatal mannequin (high-fidelity simulation)	4.5	4	5
High-fidelity simulation is beneficial to me, as it increases self-confidence	4	3	4.5
High-fidelity simulation is beneficial to me, as it reinforces theoretical knowledge	3.5	3	4
High-fidelity simulation is beneficial to me, as it enhances my communication skills and teamwork	4	4	5

a *Based on a 5-point Likert scale, where 1, strongly disagree; 2, disagree; 3, neutral; 4, agree; 5, strongly agree*.

In the open-ended comment section, participants expressed enthusiasm toward the realism and the benefits of the simulations, asking for more frequent mannequin-based sessions and wet labs to maintain the acquired skills in the long-term.

## Discussion

The delivery of a comprehensive ECMO curriculum is essential to succeed in the development of new programs. According to our findings, a multistep team training model that includes high-fidelity simulations emerged as a feasible and efficient educational tool to launch a new neonatal respiratory ECMO program. The first introductory module provided the necessary knowledge through didactic lectures. Next, the water drills allowed the trainees to better familiarize with the circuit, thus acquiring technical skills. Once theoretical and practical basis was acquired, animal-based, and high-fidelity mannequin-based simulations provided a protected environment to practice and optimize technological, cognitive and behavioral skills. In more detail, as emphasized by our pediatric surgeons, hands-on animal lab showed to be crucial to enhance cannulation ability in our surgical context. Overall, our educational model increased knowledge and self-reported confidence of novice ECMO providers. The evaluation of the team performance in relation to every single item suggests areas of strengths and limits of the training for further improvement of our ECMO curriculum, by highlighting the topics to further address versus those which emerge to be well retained by the team.

To our knowledge, this is the first report on the implementation of a multistep team simulation-based training to set-up a neonatal respiratory ECMO program, in the context of a highly-specialized *Neonatal Intensive Care Unit* environment without previous experience with cardiac surgery patients. A similar training model was previously reported in a pediatric intensive care unit, reinforcing the role of full dedicated practice, especially valuable for novice learners (18). A growing body of literature supports the benefits of the technology-enhanced simulations in medical education (20, 21), but didactical proposal varies among Centers (5, 17). Mannequin-based education is demonstrated to enhance: (1) the surgical neck cannulation (22) (2) the establishment of knowledge and confidence of novice learners across ECMO teams (18) (3) the maintenance of skills proficiency (9, 12, 14), 4. the promotion of teamwork and behavioral skills (23). Consistently, our results confirmed the benefits of the mannequin-based education, which was highly rated and appreciated by all trainees. The enthusiasm for active learning is in line with previous evidence, reporting ECMO simulations to be beneficial to learners across different health-care backgrounds and experience levels (24).

Although traditional methods of learning were associated with poor retention of knowledge and procedural skills (25), our results suggested that the conventional education maintains its role during the early steps of novice learners' training. Indeed, as previously suggested by Sanchez-Glanville et al. (18), didactic lectures may help to fill the knowledge gap, and animal-based training enhance surgical cannulation skills. Conversely, experienced ECMO providers would better benefit from active learning and, therefore, lectures series might be less suitable for a maintenance training (5).

This multistep team training was based on a multidisciplinary involvement of the members, even though masterful examples of training have been delivered to selected providers, to reach specific learning outcomes (10). ECMO emergencies require effective teamwork and communication; thus inter-professional simulations may provide the optimal setting to practice leadership and behavioral aspects (9). All the skills mentioned above are critical during an ECMO procedure, and our educational model provided an opportunity for trainees to learn in a protected environment, and to receive prompt expert feedback during the debriefing sessions. Debriefing is an essential part of the formative assessment, as it helps trainers to identify knowledge and skills' gaps and provide an individualized learning program (26). According to the results of our survey, we have critically reviewed the actual structure of debriefing sessions. We believed that video-recording of the sessions could implement the standard oral debriefing format, providing the trainees with an “outside” view of their performance (27). Moreover, the accurate portrayal of events through videotaping could support and enhance learning, by raising discussion among participants (27); however, the cost-effectiveness of a video-assisted debriefing remains unclear (28, 29).

Our study had a few limitations. Firstly, as it is a single-center study, the results cannot be generalized. Nevertheless, the training of a novice ECMO team, especially in the early steps, needs to be customized to every single center. By taking into account the center-specific background knowledge, available facilities and goals of the program, the education may be tailored to allow the optimization of a resource-intense effort. Secondly, the efficacy of our training model should be demonstrated in clinical practice. However, the primary focus of this work was to describe the implementation of a multistep team training while establishing a non-cardiac neonatal ECMO program. The evaluation of patients' outcomes was therefore beyond the scope, but we have planned to evaluate the training model and the skills maintenance over time, by repeating a trainees' assessment on a yearly basis. Thirdly, the tests used for pre- and post-curriculum competency assessment were not externally validated. Nevertheless, as they were formulated by trained ECMO physicians, according to ELSO guidelines and tailored to our clinical context, we deemed them suitable for the scope of this report. Lastly, the design of the survey questions might have posed potential response bias in favor of the training program. A more effective wording could have improved the validity of self-reported measurements (30).

## Conclusions

The multistep team training increased staff's confidence, technical expertise, and teamwork in ECMO emergency scenarios. High-fidelity simulation-based training was the most appreciated educational tool by the trainees. Although our survey suggests that the active learning is relevant for ECMO team proficiency, passive learning may maintain its role in the early establishment of the team knowledge background. Further studies should focus on the clinical impact of this educational program and the development of training and educational standards for health-providers in charge of ECMO neonates.

## Author contributions

GC, GR, SG, LP, and FM contributed conception and design of the study; GR, FCa, FCo, and CB organized the database and collected data; NP performed the statistical analysis; GR and GC wrote the first draft of the manuscript. SG provided extensive critical revision. All authors contributed to manuscript critical revision, read and approved the submitted version.

### Conflict of interest statement

FCa and FCo were employed by company Betamed Perfusion Service srl. The other authors declare that the research was conducted in the absence of any commercial or financial relationships that could be construed as a potential conflict of interest.

## Abbreviations

ECMO: Extracorporeal membrane oxygenation
ELSO: extracorporeal life support organization.
