# Supplementary material for: Start a Neonatal Extracorporeal Membrane Oxygenation Program: A Multistep Team Training
Source: Front Pediatr. 2018 May 29;6:151. doi: 10.3389/fped.2018.00151 (PMC5986935; doi:10.3389/fped.2018.00151)
Supplement: Supplementary file 1 [file Data_Sheet_1.docx]

Supplementary Material

**Start a neonatal extracorporeal membrane oxygenation program: a multistep team training**

**Genny Raffaeli^1^, Stefano Ghirardello^1^, Mara Vanzati^1^, Chiara Baracetti^1^, Francesco Canesi^1,2^, Federica Conigliaro^1,2^, Valerio Gentilino^3,4^, Francesco Macchini^4^, Monica Fumagalli^1^, Fabrizio Ciralli^1^, Nicola Pesenti^1^, Sofia Passera^1^, Simona Neri^5^, Stefania Franzini^5^, Ernesto Leva^4^, Laura Plevani^1^, Fabio Mosca^1^, Giacomo Cavallaro^1*^**

^1^NICU, Department of Clinical Sciences and Community Health, Fondazione IRCCS Ca’ Granda Ospedale Maggiore Policlinico, Università degli Studi di Milano, Milano, Italy.

^2^Betamed Perfusion Service, Rome, Italy

^3^Department of Pediatric Surgery, Ospedale Filippo Del Ponte Varese, Varese, Italy.

^4^Department of Pediatric Surgery, Fondazione IRCCS Ca’ Granda Ospedale Maggiore Policlinico, Milan, Italy.

^5^Pediatric Anesthesiology and Intensive Care Unit, Department of Anesthesia and Critical Care, Fondazione IRCCS Ca’ Granda Ospedale Maggiore Policlinico, Milan, Italy.

*** Correspondence:**

Giacomo Cavallaro

[giacomo.cavallaro@mangiagalli.it](mailto:giacomo.cavallaro@mangiagalli.it)

**Supplementary Data:**

**S1. Multistep ECMO team training: closed-ended questionnaire for knowledge assessment.**

1. You’re in charge of a neonate on VA-ECMO for a left CDH. ECMO day 3, the patient is stable. The post-oxy blood gas analysis shows: PO_2_ 150 mmHg and PCO_2_ 75 mmHg. What would you do?
2. Increase FiO_2_ and increase sweep gas at the ECMO gas blender
3. Increase respiratory rate
4. Increase sweep gas at the ECMO gas blender, maintain unchanged FiO_2_ *
5. Decrease sweep gas at the ECMO gas blender, maintain unchanged FiO_2_
6. During ECMO support, pre- and post- oxygenator pressures increase from 160-150 mmHg to 195-185 mmHg. The patient mean blood pressure increases from 40 mmHg to 70 mmHg. What is happening?
7. Due to a clot formation on the arterial cannula, the post oxygenator pressure is increased. As a consequence patient systemic pressure rises.
8. Due to systemic vasoconstriction, the circuit pressures (pre- and post- oxygenator) are increased*
9. It is not possible to sustain the blood flow
10. It is possible to think about a blood clot in the membrane lung which is compromising the ECMO performance
11. Immediately before starting ECMO, the patient activated clotting time is 110 seconds and the circuit activated clotting time is 280 seconds. Which is your next action?
12. Administer a 25 UI/kg heparin bolus to the circuit and a 25 UI/kg bolus to the patient
13. Administer a 50 UI/kg heparin bolus to the patient and go ahead with Start ECMO
14. Administer a 50 UI/kg heparin bolus to the circuit and go ahead with Start ECMO
15. Administer a 50 UI/kg heparin bolus to the patient and check patient activated clotting time*
16. ECMO day 3: the patient activated clotting time is 145 seconds, the aPTT ratio is 2, the thromboelastographic trace shows an R-K 18 min, MA 45 mm), anti-Xa is 0.3 IU/mL. Heparin infusion rate is 35 UI/kg/hr. What would you do?
17. Administer a 50 UI/kg heparin bolus to the patient
18. Get a circuit blood sample to re-check the activated clotting time value, in the meanwhile, you do not change the heparin drip*
19. Do not change in heparin drip
20. None of the above
21. ECMO day 17, ECMO blood flow is 80% of cardiac output. Pre-oxy blood gas analysis shows: PCO_2_50 mmHg; post-oxy gas analysis: PCO_2_45 mmHg; the arterial patient gas analysis shows: PCO_2_ 40 mmHg. Is it time to wean the patient?
22. Not yet, I would get a blood gas analysis from peripheral patient vein to measure venous CO_2_
23. Not yet, I think the membrane lung is not working
24. Maybe, I would reduce the support to 60% of cardiac output and repeat another blood gas analysis*
25. All of the above
26. During ECMO assistance the physician requires an RPM rise to increase the support from 70% to 90% of cardiac output. What about LPM?
27. LPM vary according to patient vascular resistance*
28. LPM does not increase, but the negative and post oxygenator pressures increase
29. It’s not possible to increase RPM because it is not the correct way to optimize support
30. LPM vary with RPM, in a directly proportional way.
31. In our ECMO asset (hemofilter is parallel with the circuit) there is a difference between pump flow and delivered flow to the patient. All of a sudden the two flows equalize.
32. The perfusionist has just clamped the line through the hemofilter*
33. The pump flow-meter does not detect the flow
34. The perfusionist has just started the haemofiltration
35. The perfusionist has clamped the post oxygenator line, thus stopping the post oxygenator flow
36. A brownout occurs during ECMO support in the intensive care unit. What happens with the ECMO machine?
37. The ECMO machine turns off, and the patient assistance is interrupted. To guarantee the flow to the patient is necessary to use the emergency unit pump
38. The ECMO machine does not turn off thanks to the internal battery, but the heat exchanger turns off *
39. The ECMO machine produces an alarm, but at the same time, it maintains the same pump flow. The heat exchanger does not turn off thanks to the internal battery
40. The ECMO machine does not produce an alarm thanks to the internal battery; the Rotaflow console's led turn off, but the pump flow is guaranteed
41. The post oxy blood gas analysis shows: PO_2_ 75 mmHg
42. Reduce FiO_2_ on the gas blender
43. Increase FiO_2_ on the gas blender*
44. Increase sweep gas in the gas blender
45. Increase both sweep gas and FiO_2_ on the gas blender
46. During Veno-Arterial start ECMO, the heparin bolus administration occurs:
47. After cannulas positioning, just before circuit connection through venous cannula
48. Before cannulas positioning, at the vessels exposure, through central or peripheral venous catheter of the patient*
49. At the beginning of the procedure, just before the vessels exposure through central or peripheral venous catheter of the patient
50. Only in the circuit, because heparin solution is continuously administered through central venous catheter of the patient
51. Your patient is on double-lumen VV ECMO for H1N1 pneumonia. ECMO day 10, stable until it begins to desaturate, suddenly after the diaper change. As you increase the blood flow, SpO2 get worse. What would you do?
52. I’ll perform echocardiography, as I’m sure it’s a spell of pulmonary hypertension
53. I’ll reduce the blood flow, as I’m fearing recirculation is occurring*
54. I’ll check the oxygenator status; maybe the membrane doesn’t work anymore
55. I’ll get a thorax X-ray to monitor the pneumonia
56. You’re going to perform a circuit replacement, what do you need?
57. Venous and arterial cannulas
58. arterial cannula
59. 1/4 -1/4 with Luer lock connector
60. 1/4 -1/4 connector*
61. a) and c)
62. Which of the following drugs should be avoided during a neonatal ECMO?
63. morphine
64. paracetamol
65. fentanyl*
66. rocuronium
67. ECMO day 10, delivering 1 L/min sweep gas on the gas blender, the pre oxy blood gas analysis shows: PCO_2_ 75 mmHg; while post oxy blood gas analysis shows: PCO_2_ 72 mmHg; patient arterial blood gas analysis shows: PCO_2_ 75 mmHg. What would you do?
68. Increase sweep gas at the gas blender and after a while repeat pre- and post- oxy and arterial patient blood gas analysis, as a decrease of both membrane lung function and native lung compliance is likely.*
69. Perform a circuit replacement due to reduced performance both in native and membrane lung gas exchange
70. Carry on with weaning because the native lung function can provide gas exchange
71. Decrease sweep gas at the gas blender and after a while repeat pre- and post- oxy and arterial patient blood gas analysis, as you’re suspecting a reduced membrane lung function and a reduced native lung compliance.
72. Which of the following has the worst survival rate after ECMO?
73. Meconium aspiration syndrome
74. Sepsis
75. Acute respiratory distress
76. Congenital diaphragmatic hernia*
77. Pneumonia
78. a) and b)
79. In which of the following clinical conditions ECMO is contraindicated?
80. Gestational age < 37 weeks
81. Intraventricular hemorrhage> III°*
82. Dilatative cardiomyopathy
83. Pneumothorax
84. All of the above
85. a) and b)
86. In the neonatal venous-arterial ECMO, which vessels are cannulated?
87. Left external jugular vein – left carotid artery
88. Right external jugular vein – right carotid artery
89. Right internal jugular vein – right carotid artery*
90. Left internal jugular vein – left carotid artery
91. All of the above
92. c) and d)
93. How does blood flow through an ECMO circuit?
94. cannula, venous line, centrifugal pump, heat exchanger, artificial lung, arterial line*
95. cannula, arterial line, centrifugal pump, artificial lung, venous line
96. cannula, venous line, artificial lung, centrifugal pump, arterial line
97. cannula, venous line, artificial lung, centrifugal pump, heat exchanger, arterial line

*correct answer
